# Plant growth, physiological variation and homological relationship of *Cyclocarya* species in *ex situ* conservation

**DOI:** 10.1093/conphys/coac016

**Published:** 2022-05-07

**Authors:** Ying Feng, Kailing Zheng, Xiulian Lin, Junpo Huang

**Affiliations:** School of Resource and Environmental Science, Quanzhou Normal University, Donghai Street, Quanzhou City, Fujian Province, 362000, China; Quanzhou Institute of Agricultural Science, Chidian Town, Jinjiang City, Fujian Province, 362000, China; Horticulture Department, Huizhou Engineering Vocational College, Xiaojinkou Street, Guangdong Province, 561023, China; School of Resource and Environmental Science, Quanzhou Normal University, Donghai Street, Quanzhou City, Fujian Province, 362000, China

**Keywords:** physiological index, leaf morphology, homological relationship, *ex situ* conservation, *Cyclocary paliurus*

## Abstract

Natural forests of *Cyclocarya paliurus* have been seriously damaged because of the extreme demand for leaf medicinal uses, making conservation of this valuable, medicinal woody species necessary. Because of geographical differentiation and diverse adaptability, in this study we analysed the variations in plant growth and physiological response to environmental factors at a resource plantation of *ex situ* conservation and determined the homological relationships between local provenance (from Fujian Province, FJ) and introduced provenances showing high-survival rate and better growth (from Zhejiang, Hubei, Guizhou and Jiangxi Province). Our results suggested the following: (i) Plant growth: FJ had the highest plant height but not the largest basal diameter in comparison to that of other provenances. (ii) Physiological responses during the growth periods: water content in leaf of FJ had similar change with that of other provenances, except for the provenance from Guizhou Province; total soluble sugar content in leaf of FJ was more than that of other provenances; calcium content in leaf of all provenances was higher as compared to K, Mg and Na; the highest activity among four kinds of antioxidant enzymes in all provenances was superoxide dismutase, then was polyphenol oxidase and peroxidase, finally was catalase; and total flavonoid among three kinds of secondary metabolites in all provenances showed the greatest content, followed by polysaccharides and total triterpenoid. (iii) Relation analysis: plant growth and physiological responses related with environmental factors, especially temperature and precipitation. (iv) Homological relationships: leaf characteristics among six provenances varied in colour, area and common petiole length, but not the shape of leaf base or apex. *Cyclocarya paliurus* distributed in Fujian Province showed a very close homological relationship with that distributed in Zhejiang Province by simple sequence repeat. These findings will provide knowledge on physiological response to environmental factors and aid to select suitable provenances for *Cyclocarya* cultivation.

## Teaser

It is necessary to conserve *Cyclocarya* species, as it is a valuable, medicinal woody species. *Cyclocarya paliuru*s responded to varying environmental factors via physiological changes and exhibited variation in growth at a resource plantation of *ex situ* conservation. The homological relationships revealed low genetic differentiation with only two major sub-populations.

## Introduction

Forests are a major natural resource, but with the rapid growth of human population and industrialization, massive forest destruction has occurred that is much beyond regeneration, mainly because of over-exploitation, overgrazing, unsustainable practices, forest fires and environmentally unfriendly development projects (Babu and Nautiyal, 2015). Therefore, conserving forest resources, especially high-value species and those with small and vulnerable populations, is pertinent for genetic resource conservation (Holliday *et al.*, 2017; Ratnam *et al.*, 2014). *Cyclocarya paliurus* (Batal.) Iljinskaja (*C. paliurus*) is a native and high-value species distributed in the highlands of sub-tropical areas in China (Fang *et al.*, 2006) that possesses a myriad of human health benefits, such as anticancer, antimicrobial, antihyperlipidemic, antioxidant and anti-inflammatory effects, which is primarily the result of the biological activities of various phytochemicals in their leaves (Xie *et al.*, 2012, 2013; Wang *et al.*, 2017; Liu *et al.*, 2018a, b; Shang *et al.*, 2018; Xiong *et al.*, 2018). However, *C. paliurus* regenerates slowly in natural forests because of their high seed dormancy under natural conditions (Fang *et al.*, 2006); further, the populations of *C. paliurus* has been subjected to severe damage due to the increasing medicinal use of leaves in recent years. To date, *C. paliurus* has been protected via different conservation statuses, including critically endangered, server convention and convention (http://www.iplant.cn). Therefore, it is highly pertinent to establish an effective way to conserve *C. paliurus* populations.

*Ex situ* conservation is an effective way to preserve plant species in order to rescue or maintain the natural plant biodiversity (Corlett, 2016; Seaton *et al.*, 2010). Seed banks and other biotechnological technologies, such as *in vitro* culturing, are unsuitable for conserving *C. paliurus* populations, because of their high rate of ‘empty seed’ and limitations of the *in vitro* regeneration system (Fang *et al.*, 2006; Feng *et al.*, 2020a). Thus, *ex situ* conservation could be a suitable method to conserve *C. paliurus* populations.

During the process of *ex situ* conservation, environmental factors affect plant growth and adaptation (Enßlin *et al.*, 2011; Cao *et al.*, 2018). Specifically, studies have indicated that temperature accelerates *Larix chinensis* or *Myrsine seguinii* growth (Liu *et al.*, 2018a; Wu *et al.*, 2019), but inhibits olive growth (Benlloch-González *et al.*, 2016). Plants respond to changing environmental factors by change in water content (WC) or soluble sugar content (Ben Abdallah *et al.*, 2017; Feng *et al.*, 2020b; Wu *et al.*, 2018), by regulating mineral element concentrations (Wu *et al.*, 2019) or by increasing antioxidant enzyme activities (Habibi, 2017; Zhao *et al.*, 2018). Wu *et al.* (2018) found that a change in leaf K concentrations can affect leaf water potential in response to warming. However, plants respond to environmental changes in a species-specific manner (Wu *et al.*, 2019). In addition, as a characteristic of physiological response, the accumulation of secondary metabolites is also affected by environmental factors. For example, light and fertilization influence the growth and total flavonoid accumulation of *C. paliurus* (Deng *et al.*, 2012; Yang *et al.*, 2017; Liu *et al.*, 2018a, b). Physiological responses can ultimately lead to differences in growth and adaptation among various plant species, thereby determining whether the establishment of *ex situ* construction is successful and valuable. Thus, understanding the physiological responses to environmental factors is critical for the successful construction of a resource plantation.

In addition, during long-term natural evolution processes, genetic differentiation of *C. paliurus* has occurred in natural forests via natural or human selection, including genetic drift, climate change and seed dispersal. Therefore, the relationship among *Cyclocarya* populations must be strictly defined in the *ex situ* conservation. Simple sequence repeat (SSR) has the advantage of being abundant and low-copy among the transcribed fractions of plant genomes (Uncu and Uncu, 2020) and thus has been extensively applied to analyse homological relationships in several plants, including sweet cherries (Liang *et al.*, 2018; Patzak *et al.*, 2020), peaches (Dettori *et al.*, 2015), lemons (Zhu *et al.*, 2016) and hazelnuts (Bhattarai and Mehlenbacher, 2017). Suvi *et al.* (2020) investigated the homological relationship and genotypic structure of 54 rice accessions using SSR to select unique parents for breeding. Li *et al.* (2017) used SSR to analyse the homological relationship of *C. paliurus* populations from 26 provenances of 11 provinces in China. However, the homological relationship among *C. paliurus* populations from some province is still unknown, such as the population from Fujian Province. Hence, the definition of the homological relationship of *C. paliurus* populations will further our understanding and provide essential information to enable more efficient use of available genetic resources (Mohammadi and Prasanna, 2003).

Since 2014, a resource plantation of *ex situ* conservation has been gradually established in the Fujian Province by collecting seeds from different natural populations of *C. paliurus*. However, many provenances of *C. paliurus* have not survived or show weak growth, suggesting that environmental factors affect the growth and adaptation of *C. paliurus* populations. However, to date, plant growth and physiological responses to environmental factors have received little attention during *ex situ* conservation. Therefore, this study analysed (i) variations in plant growth between local provenance (from Fujian Province) and introduced provenances showing high survival rate and better growth at the resource plantation on the basis of continuous observation and investigation over 3 years and (ii) physiological responses of six *Cyclocarya* provenances to environmental factors and (iii) defined the homological relationships among six *Cyclocarya* provenances at the resource plantation. This could provide new knowledge on physiological response to environmental factor and homological relationship at *ex situ* conservation of *C. paliurus* and benefit to select suitable provenances for *Cyclocarya* cultivation.

## Material and methods

### Plant materials and site description

In this study, 1-year-old plants of *C. paliurus* were conserved in Xiayang Town, Quanzhou City, Fujian Province (25°19′19″N, 118°17′39″E; mean annual temperature, 20 ± 2°C; annual precipitation, 1300–1500 mm; annual sunlight, 4200–4800 h). Plants were grown from seeds collected from the following six provenances: Yongchun (FJ) Fujian Province, Anji (AJ) Zhejiang Province, Wufeng (WF) Hubei Province, Tonggu (TG) Jiangxi Province, Jinggangshan (JX) Jiangxi Province and Jianhe (JH) Guizhou Province, and environmental factors on *C. paliurus* of the six provenances can be seen in Supplementary Information Table S1. In total, 50 plants of each provenances were planted in each block with a spacing of 2 × 2 m. Artificial weeding was performed in May and September every year, but no supplemental irrigation was given for plant growth. Climatic factor data during the experiment period at the experimental site were collected from a local weather bureau, and all indices were summarized in Table 1. The soil at the experimental site was sandy loam soil containing 5.64 ± 0.06 (mg/kg) available nitrogen, 16.28 ± 0.06 (mg/kg) available phosphorous and 4.45 ± 0.05 (mg/kg) available potassium.

**Table 1:** Environmental parameters in the *ex situ* conservation site

Indices	Year			Indices	Month			
	2017	2018	2019		April^a^	June^a^	August^a^	October^a^
MAT (°C)	20.6	20.4	18.6	T (°C)	18.4	23.5	24.7	18.1
AP (mm)	1296.4	1794.7	1570.7	P (mm)	64.3	227.7	353.7	53
AS (h)	1874.6	1825.6	1600.6	S (h)	152	123.8	177	159.2

a Climatic parameters were recorded in 2018. MAT, mean annual temperature; AP, annual precipitation; AS, annual sunlight; T, average temperature; P, precipitation; and S, sunlight.

After 1 year *ex situ* conservation, leaf samples were collected from the middle part of the current-growth branch in April, June, August and October. The collected leaves were maintained at 4°C and then immediately transported to the laboratory. Some leaves were dried for analysing physiological changes in WC, mineral concentration and secondary metabolite accumulation; other leaves were immediately frozen in liquid nitrogen and stored at −80°C for analysing total soluble sugar (TTS) content and antioxidant enzyme activity. Meanwhile, leaves collected in August were analysed for homological relationship according to leaf morphological characteristics and SSR analysis.

### Plant growth determination

In April, all plants were observed and the initial height (H_i_) and initial basal diameter (BD_i_) were measured. Then, the plant height (H_a_) and basal diameter (BD_a_) of all the surviving plants were measured again in December. Continuous measurements were conducted from 2017 to 2019. The growth index was calculated as follows:

The height increase of plant: (NH) = H_a_– H_i_.

The basal diameter increase of plant: (NBD) = BD_a_—BD_i_.

The average increase of plant height: the sum of NH/the number of plants.

The average increase of basal diameter: the sum of NBD/the number of plants.

### Leaf physiological determination

#### Determination of WC

According to the method described by Stein *et al.* (1975), WC in leaf was calculated as WC (%) = (FW − DW)∕FW × 100, where FW is the weight of the fresh leaf, and DW is the constant weight of the dried leaf.

#### Extraction and determination of TTS content

TSS was extracted with distilled water from the fifth and sixth leaflets of the fresh compound leaf using the Anthrone-H_2_SO_4_ method described by Li (2000), then the absorbance at 630 nm was measured with a UV-visible spectrophotometer, finally TSS content was calculated as follows: leaf TSS content (%) = (C × 25)/(W × 0.5 × 10^6^) × 100, where C is obtained from the standard curve constructed with sugar and W is the weight of the fresh sample.

#### Extraction and determination of mineral nutrients

Samples were digested using the electric-heating digestion method described by Feng *et al.* (2020b). The content of mineral nutrients [potassium (K), sodium (Na), calcium (Ca), and magnesium (Mg)] were calculated using the following equation: (C × 0.025)/DW, where C is mineral content measured via Inductively Coupled Plasma Optical Emission Spectrometer (ICP-OES, Optima 7000DV, USA) and DW is the weight of dried sample.

#### Extraction and determination of antioxidant enzyme activity

Antioxidant enzyme extraction was obtained and each activity of four antioxidant enzymes [superoxide dismutase (SOD), polyphenol oxidase (PPO), peroxidase (POD), catalase (CAT)] was analysed in accordance with the method of Feng *et al.* (2020b).

#### Extraction and determination of secondary metabolite accumulation

Extraction was performed using an ultrasonic-assisted method with slight modifications (Liu *et al.*, 2018c). Briefly, each sample (~1.0 g) was added to 20 ml of 75% ethanol, centrifuged at 25°C and 11 000 *g* for 15 min after heating at 70°C for 60 min with an ultrasonic cleaner (KQ-800DE, China).

Total flavonoid content was determined using the method described by Liu *et al.*, (2018c). A standard curve was constructed with rutin, and total flavonoid content was expressed as mg rutin equivalent/g dry mass.

Total triterpenoid content was assessed using the Folin–Ciocalteu colourimetric method and then expressed as mg gallic acid equivalent/g dry mass.

Polysaccharide content was determined using the method described by Liu *et al.*, (2018a). A standard curve was constructed with glucose, and polysaccharide content was expressed as mg glucose equivalent/g dry mass.

### Homological relationship definition

#### Leaf morphological determination

Changes in leaf colour and leaf shape were observed, and total leaf area and the length of common petioles were measured for leaf samples from local provenance and five introduced provenances at resource plantation. Five compound leaves from the same plant were measured and treated as one leaf sample. And the leaf measurement of each provenance consisted of three replicates, with 10 leaf samples per replication.

Leaf cross sections were cut into ~1 × 1 cm segments from the middle part of the fresh leaves for each provenance. The sections were fixed in 2.5% glutaraldehyde, soaked in osmic acid for 1 h and then dehydrated using a graded ethanol series (100,0, 75:25, 50:50, 25:75 and 0:100, v/v), for which samples were kept at each concentration for 15 min. The sections were critical-point dried in carbon dioxide using a critical point dryer (Leica EM CPD300, Germany), coated with gold–palladium using a vacuum coater (Leica EM ACE200, Germany) at 15 mA, viewed and photographed with a scanning electron microscope (SEM; FEI Quanta450, USA).

#### DNA extraction

DNA was extracted from fresh leaves of the six provenances using the improved Hexadecyl trimethyl ammonium Bromide (CTAB) method. Each sample was added to CTAB solution (100 mmol/l NaCl, 20 mmol/l Ethylene Diamine Tetraacetic Acid (EDTA) (pH 8.0), 2% CTAB (w/v) and 100 mmol/l Tris–HCl), heated at 65°C for 30 min and centrifuged at 12000 *g* (TGL-16G, China) at 25°C for 5 min. The supernatant was obtained and added to phenol-chloroform (1:1 v/v), then centrifuged at 12000 *g* at 25°C for 10 min. Next, the supernatant was again obtained, added to chloroform and finally centrifuged at 12000 *g* at 25°C for 10 min. This step was repeated twice. Finally, the supernatant was added to isopropanol, kept at room temperature for 15 min and centrifuged at 12000 *g* at 25°C for 6 min. The deposit was cleaned with 75% ethanol and dissolved in 50 μl TE. Extracted DNA samples were stored at −20°C.

#### S‌SR amplification

A total of 50 ng/μl DNA was used for polymerase chain reactions (PCRs) via a PCR analyser. Ten pairs of SSR primers were selected from Fan *et al.* (2013) and used for SSR amplification (Table 2). The PCRs were conducted in a 20-μl reaction containing 2.0 μl 10 × Buffer, 0.1 mmol/l dNTPs, 0.3 μmol/l of each primer and 1.0 U *Taq* DNA polymerase. SSR amplification was performed using the following conditions: initial denaturation at 95°C for 5 min, followed by 37 cycles at 94°C for 30 s, an appropriate annealing temperature (from 65°C to 55°C using a graded decreasing temperature from the first to the tenth cycle, then cooling from 55°C from cycles 11 to 37) for 35 s, 72°C for 40 s and finally extension at 72°C for 3 min. The SSR products were separated into a 6% denatured polyacrylamide gel and stained using the silver staining protocol.

**Table 2:** Characteristics of SSR primers used in the present study

Primer	Primer sequences (5′–3′)	Repeat motif	Allele size (bp)	Na	Ne	He	I	Npl	Tl	Ppl (%)
S1	F: ACCCCTCAAGTCCCACCA R:CCAGATACACATGCACAC	(CT)_11_	178	1.80	1.31	0.21	0.33	4	5	80
S2	F:ATTCCCCACCCCCATCTC R:CTCCTCCAGCGCACATAA	(CT) _8_	201	2.00	1.56	0.34	0.52	8	8	100
S3	F:ATCGTCCTGGTG ATGTTG R:AGGTCCTCCTTCCTTTGG	(AC)_7_	167	1.67	1.21	0.15	0.25	2	3	66.67
S4	F:TGCCTCAATCCCAAAGAC R:AATTACGCCGAAGGGGTC	(TG)_7_ (AG)_7_	208	2.00	1.33	0.22	0.37	5	5	100
S5	F:AGATGGCTTTTCAGATTTG R:CGGAAACTTGAATCAGAG	(CT) _12_	105	1.89	1.54	0.31	0.47	8	9	88.89
S6	F:GCTGATGGTAATGGTTTTTAG R:ACAAAACCGACTGACAACAA	(CT)_10_(CTCTGT)_5_	175	2	1.38	0.26	0.43	14	14	100
S7	F:ACCCAAAAGAAAAGCA R:CGGTGAAATCTACTCCAA	(AG)_6_AA(AG)_7_	103	2	1.35	0.24	0.39	11	11	100
S8	F:AGCCACCGCTAGGAAGCA R:GGGCGTTACAGTGGGAGA	(CT)_12_	122	2	1.53	0.33	0.51	8	8	100
S9	F:TCCTCCACTTCCAATGAT R:AGAGGAGCAAACAAACAT	(CT)_17_	196	2	1.33	0.23	0.38	11	11	100
S10	F:AGAGATTAGCTCGGGTCT R:GATCCAAAACTGAAGGGA	(TG)_13_(AG)_15_	126	2	1.42	0.28	0.44	12	12	100
Mean				1.97	1.41	0.27	0.43	8.3	8.6	96.51

Na, number of alleles/locus; Ne, effective number of alleles; He, Nei’s genetic diversity; I, Shannon’s information index; Npl, total number of polymorphic loci; Tl, total number of loci; and Ppl, percentage of polymorphic loci.

#### Data and statistical analysis

One-way analysis of variance was used to test significant differences in plant growth from 2017 to 2019 and leaf physiological indices during the growth period by SPSS statistical software package (Version 16.0, IBM, USA) on the basis of Tukey’s Highly Significant Differences at the significance level of *P* < 0.05. Pearson Correlation Analysis and trend-surface analysis by the performance of Origin software 9.1 (Northampton, MA01060, USA) was used to analyse plant growth and leaf physiological indices in correlation with environmental factors. Principal Component Analysis (PCA) and Homology Analysis were also performed by Origin software 9.1 to analyse the relation between different physiological indices.

An Excel original binary data matrix was constructed by calculating the presence-absence data of each amplified fragment, and these data were analysed using NTSys v.2.10 (Applied Biostatistics, Setauket, NY, USA). The genetic index of the number of alleles/locus (Na), effective number of alleles, number of polymorphic loci, percentage of polymorphic loci, Nei’s gene diversity (He) and Shannon’s information index (I) were also calculated using POPGen32. The generation of a clustering graph analysis among the six provenances was performed using NTSys v.2.10.

## Results and analysis

### Variable plant growth among the six *Cyclocarya* provenances

Plant height and BD grew faster after 1-year *ex situ* conservation and significantly increased with the long-term conservation. Both of plant height and BD in 2019 reached significance as compared to that in 2017 and in 2018 (Table 3). However, plant growth varied with provenances within a year. Specifically, FJ had the highest plant height and the increase in plant height showed the following order: FJ > WF > JH > TG > JX > AJ; nonetheless, JH had the largest BD. The increase in plant basal diameter ranked as follows: JH > FJ > AJ > JX > WF > TG up to 2019.

**Table 3:** Variations in plant growth of *C. paliurus* among six provenances

Index	Year	FJ	JX	WF	TG	JH	AJ
H	2017	10.67 ± 3.34b	15.70 ± 1.51c	7.08 ± 0.79c	18.00 ± 4.47c	18.98 ± 1.53b	10.71 ± 0.29c
	2018	48.15 ± 16.46b	88.75 ± 6.51b	100.06 ± 7.05 b	59.42 ± 2.98b	72.32 ± 12.19b	72.57 ± 15.50b
	2019	300.17 ± 29.33a	148.33 ± 18.00a	269.16 ± 28.13a	168.29 ± 14.48a	236.82 ± 47.19a	133.58 ± 27.42a
BD	2017	3.39 ± 1.27b	2.56 ± 0.79c	1.62 ± 0.29c	1.54 ± 0.45c	1.80 ± 0.22b	1.11 ± 0.14c
	2018	7.92 ± 4.24b	9.07 ± 1.76 b	10.36 ± 1.88b	8.13 ± 1.20b	9.74 ± 1.61b	9.03 ± 1.81b
	2019	27.12 ± 3.73a	21.84 ± 1.49a	20.49 ± 4.52a	19.78 ± 1.63a	53.19 ± 5.45a	25.27 ± 1.09 a

H, average increase of plant height; BD, average increase of plant basal diameter.

### Analysis of variability in physiological performance

#### Water content

**Figure 1:**
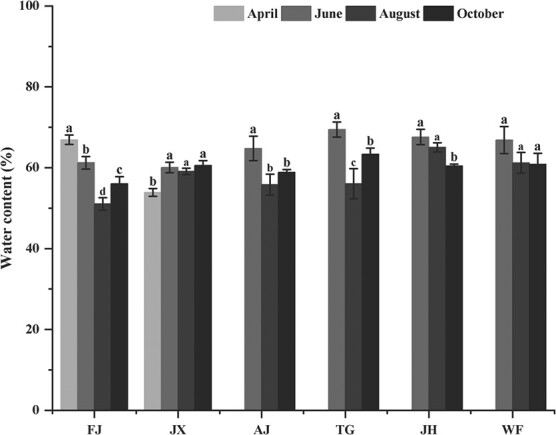
WC in leaves of *C. paliurus* of the six provenances: FJ, JX, AJ, TG, JH and WF,.

Leaves of FJ had the highest WC and reached significance, but those of JX showed the lowest WC in April, compared to that in other month. WC in leaf of FJ, AJ, TG, JX and WF showed similar changes from June to October, reaching minimum in August. In particular, the WC of JH decreased gradually after reaching the highest content in June, but no difference in leaf WC was observed among in June, August and October (Fig. 1).

#### T‌TS content

**Figure 2:**
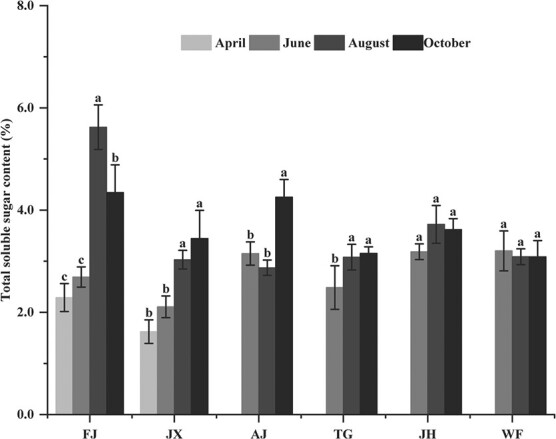
TTS contents in leaves of *C. paliurus* of the six provenances: FJ, JX, AJ, TG, JH and WF.

TSS content in leaf of FJ was more than that of JX in April. TSS content in leaf of FJ and JH increased firstly and then decreased from June to October and reached the highest value in August, but there was no difference in TSS content in leaf of JH among in June, August and October. The change of leaf TSS content of AJ was opposite to the behaviour of FJ and JH. Meanwhile, TSS content in leaf of JX and TG increased gradually, reaching significance in October, which was contrary to that of WF with the decreasing TSS content from June to October (Fig. 2).

#### Mineral nutrient content

**Figure 3:**
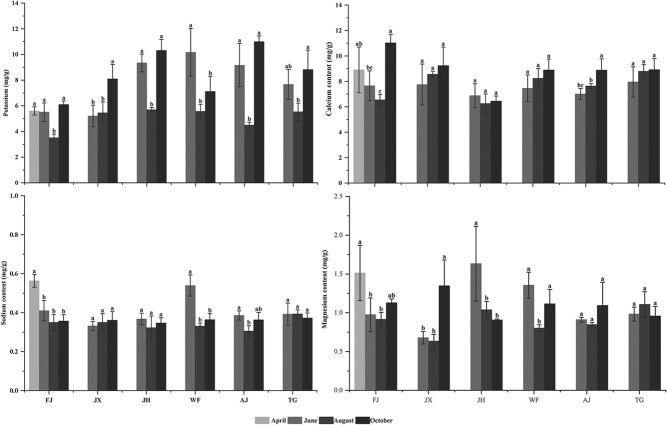
Mineral accumulations in leaves of *C. paliurus* of the six provenances: FJ, JX, AJ, TG, JH and WF.

K, Ca, Na and Mg were present in the leaves of all six provenances, in which the highest mineral content was Ca (≥6.0 mg/g), followed by K (≥3.0 mg/g) and Mg and Na (≤2.0 mg/g) (Fig. 3).

For the six provenances, K content decreased firstly and then increased, having the lowest content in August. Similarly, Ca content in leaf of FJ and JH decreased firstly and then increased, but leaf of JX showed an increasing content of Ca and a similar change was observed in that of WF, AJ, TG and JX. The change of Mg content in leaf of FJ, JX, WF and AJ decreased firstly and then increased, which was contrary to that of TG. Conversely, leaf of JH had the decreasing content of Mg. Leaf of FJ, JH, WF and AJ had similar change of Na content, while leaf of JX and TG had the increasing content of Na.

#### Antioxidant enzyme activity

**Figure 4:**
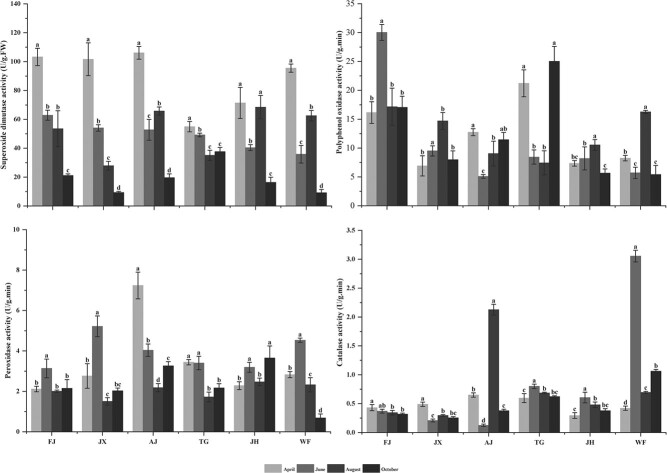
Variations in antioxidant enzyme activity of *C. paliurus* of the six provenances: FJ, JX, AJ, TG, JH and WF.

The four antioxidant enzyme activities in leaf of the six provenances decreased in the following order: SOD > PPO > POD > CAT. However, the enzymes showed different changes in activity from April to October (Fig. 4).

In particular, SOD activity in leaf of the six provenances in April decreased in the following order: AJ > FJ > JX > WF > JH > TG. SOD activity in leaf of FJ decreased gradually from April to October, and similar changes were observed in leaf of JX and TG. Conversely, SOD activity in leaf of WF, AJ and JH decreased firstly, then increased, and finally decreased again.

PPO activity in leaf of FJ was 30.04 U/(g·min) and revealed a significant difference in June, while leaf of AJ exhibited an opposite behaviour in PPO activity with that of FJ. Both of JX and JH showed similar changes in PPO activity, peaking in August. Finally, PPO activity in leaf of TG and WF reached maximum in October and August, respectively.

Different peaks of POD activity were observed in leaf of the six provenances. Specifically, both JX and FJ showed a PPO activity greater than 3.0 U/(g·min) in June, while TG and JH had the highest activity in April and October, respectively. There were similar change in PPO activity in leaf of FJ, JX, TG and JH, but leaf of AJ exhibited an opposite change in PPO activity with that of WF.

CAT activity in leaf of JX and FJ were less than 0.5 U/(g·min) from April to October. A similar change was found in leaf of TG and JH, although the activity in leaf of TG was higher than that of JH. Meanwhile, leaf of AJ and WF reached maximum CAT activity in August and June, respectively, and the peak was more than 2.0 U/(g·min).

#### Analysis of secondary metabolite accumulation

**Figure 5:**
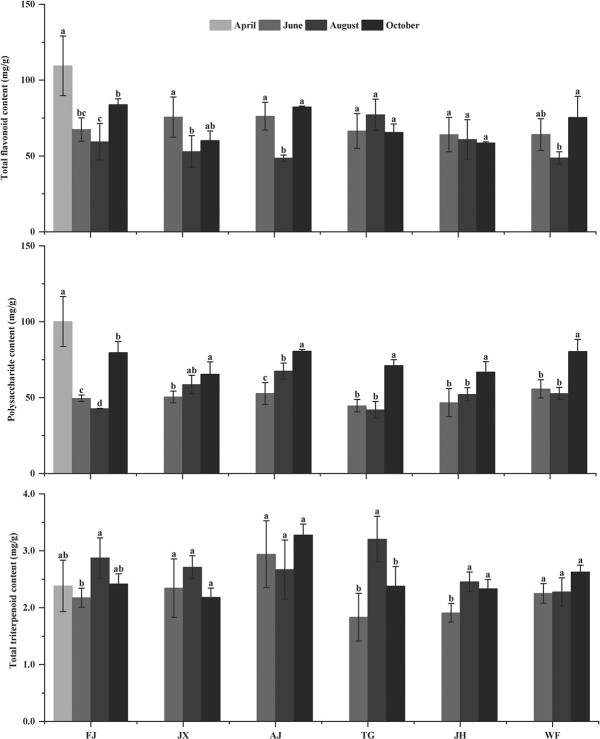
Variations in secondary metabolite accumulation of *C. paliurus* of the six provenances: FJ, JX, AJ, TG, JH and WF.

High contents of total flavonoid and polysaccharides (≥40 mg/g) were detected, but total tritenpenoid content was low (≤3.0 mg/g) in leaf of the six provenances. However, the accumulation of three kinds of secondary metabolite in leaf showed different changes from April to October (Fig. 5).

Total flavonoid content in leaf of FJ decreased firstly and then increased from April to October, which was similar to that of JX, WF and AJ. However, there was no significant difference in total flavonoid content observed in leaf of JH and TG from June to October.

Polysaccharide content in leaf of FJ reached 100.16 mg/g in April, and then decreased by 50.52%, 57.29% and 20.39% in June, August and October, respectively. The other five provenances had the highest content of polysaccharides in October, while both of WF and AJ had similar change in polysaccharides accumulation and similar change exit between that of JH and TG.

The highest content of total triterpenoid in August was observed in leaf of FJ, JX, JH and TG, all of which exhibited similar change, but were contrary to that of WF and AJ. Leaf of AJ showed no significant difference in triterpenoid concentration from June to October, and similar results were observed in leaf of WF and JX.

### Analysis of homological relationship

#### General morphological differences in the leaves

Leaf colour of FJ changed gradually from red to green, and similar changes were observed in leaf of AJ. However, there was no change in leaf colour of TG, WF, JX and JH (Table 4).

**Table 4:** Leaf characteristics of *C. paliurus* among six provenances

Code	Leaf colour	Shape			Leaf sum	Leaf area (cm^2^)	Common petiole length (cm)
		Leaf	Leaf base	Leaf apex			
WF	Green	Long elliptic	Skewness	Acute	10 ± 2 bc	205.05 ± 2.76 b	17.81 ± 3.09 b
TG	Green	Broad elliptic	Skewness	Acute	9 ± 2 c	227.92 ± 16.45 a	19.46 ± 0.38 ab
JX	Green	Broad elliptic	Skewness	Acute	9 ± 1 c	233.91 ± 9.04 a	20.18 ± 3.50 ab
JH	Green	Long elliptic	Skewness	Acute	11 ± 2 b	225.59 ± 11.29 a	22.97 ± 0.70 a
AJ	Red-green	Long elliptic	Skewness	Acute	11 ± 1b	196.20 ± 14.08 c	19.95 ± 1.79 ab
FJ	Red-green	Long elliptic	Skewness	Acute	13 ± 1 a	184.86 ± 10.95c	21.91 ± 0.29 a

Moreover, leaf shape was similar among FJ, AJ, WF and JH. Further, there were no differences in the shape of leaf apex and leaf base among the six provenances (Table 4).

Additionally, there were also differences in leaf number, leaf area and petiole length among the six provenances. FJ had the highest number of simple leaves, reaching significance with other provenances. Leaf area of FJ was the lowest and had significant difference with other provenances, except for that of AJ. The common petiole length of JH was the longest, but there was no difference in that of FJ and JH (Table 4).

#### SEM observation on the leaf surfaces

**Figure 6:**
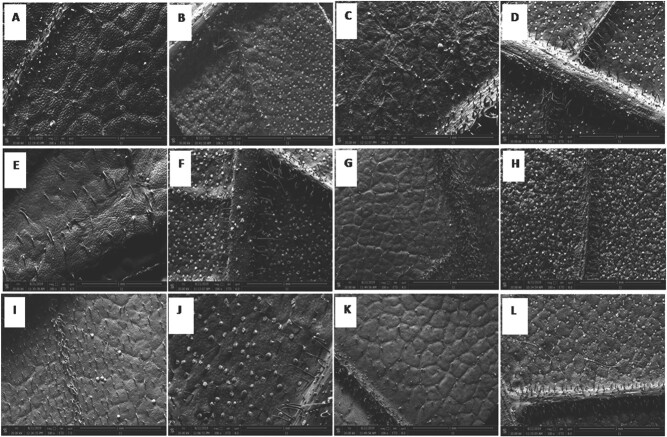
SEM images of leaves from *C. paliurus* of the six provenances: FJ, JX, AJ, TG, JH and WF. A, C, E, G, I and K represent the upper surface of a leaf from TG, WF, AJ, FJ, JX and JH, respectively. B, D, F, H, J and L indicate the lower surface of a leaf from TG, WF, AJ, FJ, JX and JH, respectively.

Leaf of the six provenances had similar trichomes and stomata. However, the veins on the upper surface of the WF leaf were swollen, which was different from the others. In addition, more nectaries were observed in leaf of FJ and AJ in comparison to that of the other provenances (Fig. 6).

#### Allelic information based on SSR

A total of 86 alleles were detected with 10 pairs of SSR primers, of which 3–14 alleles were amplified among the primers, with an average of 8.6 (Table 2). The highest number of alleles was 14 by S6, whereas 12 alleles were revealed by S10 (Table 2). The mean percentage of polymorphic loci was 96.51%.

The number of alleles scored/locus ranged from 1.67 to 2 with a mean of 1.97. Meanwhile, the Ne/locus varied from 1.21 to 1.56, with a mean of 1.41, of which S2 had the highest number of effective alleles. The mean values of He and I were 0.27 and 0.43, respectively (Table 2).

#### Homology among the six provenances of *C. paliurus*

**Figure 7:**
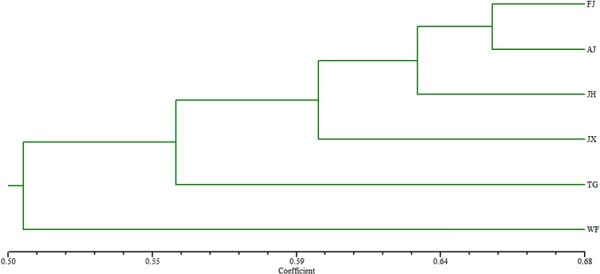
Homology relationships of the six *Cyclocarya* provenances: FJ, JX, AJ, TG, JH and WF.

Among the six provenances, two groups were clustered with a coefficient of 0.51. In particular, group 1 contained only one provenance (WF), while group 2 included five provenances (FJ, AJ, JH, JX and TG), with FJ and AJ further clustering into one subgroup (Fig. 7).

## Discussion

### Relationship between environment factors and plant growth

**Figure 8:**
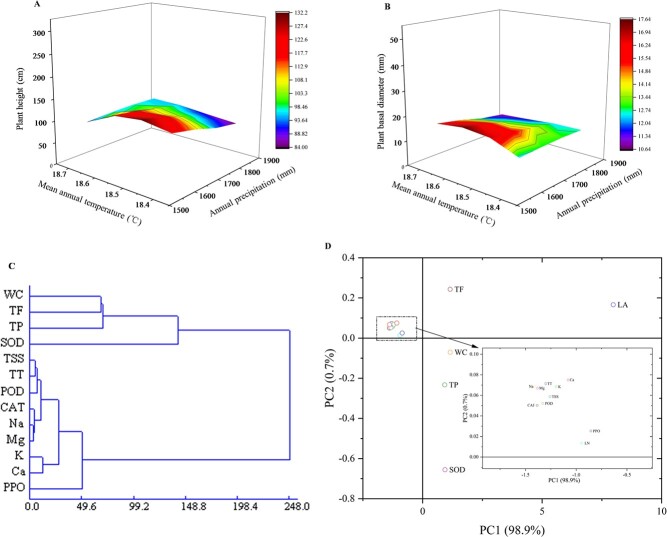
Relation analysis between environmental factors and physiological parameters. (**A**) Trend analysis between plant height and mean annual temperature and annual precipitation. (**B**) Trend analysis between plant basal diameter and mean annual temperature and annual precipitation. (**C**) Cluster analysis among various physiological indices. (**D**) PCA among various physiological indices.

Plant growth is influenced by various environmental factors (Enßlin *et al.*, 2011; Benlloch-González *et al.*, 2016; Cao *et al.*, 2018; Liu *et al.*, 2018a; Wu *et al.*, 2019), further demonstrated from our trend-surface analysis (Fig. 8A and B), but which was contrary to a previous study in which Deng *et al.* (2015) indicated that environmental factors were not correlated with the height increase of *C. paliurus*. These inconsistent findings could be explained by the species-specific response of plant growth to environmental factors (Wu *et al.*, 2019). Further, geographical differentiation developed in the *Cyclocarya* species, and the plants reported in Deng *et al.* (2015) were from Shucheng, Anhui Province (31°02′N, 116°32′E), Anji (AJ), Zhejiang Province (30°41′N,119°41′E), Lushan, Jiangxi Province (29°33′N, 116°30′E), Hefeng, Hubei Province (29°48′N, 110°11′E), Kunming, Yun-nan Province (25°02′N, 102°44′E) and Jianhe, Guizhou Province (26°31′N,108°42′E) and experimental site was in Anhui, all of which was different from the plants and experimental site in our report.

### Physiological responses of plants to environment factors

Plants respond to changeable environment factors by a series of physiological activities (Habibi, 2017; Wu *et al.*, 2018; Zhao *et al.*, 2018; Wu *et al.*, 2019; Feng *et al.*, 2020b) as further demonstrated by our findings (Table 5, Fig. 8D). As essential substrates for plant growth, WC or TSS in leaf varied with the growth period (Figs 1 and 2) and related with environment factors (Table 5), suggesting they could take part in many biosynthetic processes for improving plant adaptation (Ben Abdallah *et al.*, 2017; Wu *et al.*, 2018). As essential nutrients for plant growth, mineral elements (K, Ca, Mg, and Na) play an important role in physiological functions, such as osmotic adjustments, water balance, water use efficiency improvement and stomatal control (Coskun *et al.*, 2013; Gattward *et al.*, 2012). A correlation analysis showed that K, Ca, Mg and Na were negatively correlated with temperature and precipitation (Table 5), suggesting that plants respond to varying environments by regulating their mineral element concentrations (Wu *et al.*, 2018; Wu *et al.*, 2019). In addition, plants have developed antioxidant defence systems to adapt to changing environmental factors during the growth period. As enzymatic defence mechanisms, antioxidant enzymes, such as SOD, CAT, PPO and POD, are expected to cope with the harmful effects of reactive oxygen species (ROS) and eliminate excessive H_2_O_2_ and O_2_ in the plant tissues (Burducea *et al.*, 2019; Gill and Tuteja, 2010; Tang *et al.*, 2012). Our results also showed that SOD activity in leaf of all provenances was the highest among the four examined antioxidant enzymes and was related to environmental factors during the growth period (Fig. 4 and Table 5), inferring that SOD might be the main enzyme to interfere with the accumulation of ROS and to metabolize excess ROS produced under environmental stress (Burducea *et al.*, 2019; Gill and Tuteja, 2010).

According to our results, *C. paliurus* leaves were rich in flavonoids and polysaccharides, which was consistent with previous studies (Fang *et al.*, 2011; Xie *et al.*, 2012; Yang *et al.*, 2017; Liu *et al.*, 2018a, b; Shang *et al.*, 2018). However, total flavonoid content in *C. paliurus* was greater than that reported in previous studies (Liu *et al.*, 2018a, b; Zhou *et al.*, 2019), whereas both of polysaccharide and total triterpenoid concentrations were lower than those previously reported in Deng *et al.*, (2017) and Zhou *et al.* (2019). This may be because of differences in the extraction method: the extraction soluble used in our study was 75% ethanol, while that used in previous studies was water.

Variation in secondary metabolite accumulation is also influenced by environmental conditions (Liu *et al.*, 2018a, b; Zhou *et al.*, 2019). For example, Djerrad *et al.* (2015) reported that environmental conditions have an important effect on the essential oils in *Pinus halepensis*. Further, previous reports (Deng *et al.*, 2015; Fang *et al.*, 2011; Liu *et al.*, 2018a, b) indicated that the growth environment significantly affected secondary metabolite accumulation in *C. paliurus*, which is in accordance with our findings (Table 5).

**Table 5:** Correlation between environmental factors and physiological index

Factors	T	P	S	WC	TSS	SOD	CAT	POD	PPO	K	Na	Ca	Mg	TP	TF	TT
T	1															
P	0.97^**^	1														
S	−0.01	0.23	1													
WC	−0.04	−0.19	−0.62^**^	1												
TSS	−0.11	0	0.44	−0.60^**^	1											
SOD	0.43	0.41	−0.07	0.25	−0.35	1										
CAT	0.21	0.18	−0.09	0.15	−0.1	0.02	1									
POD	0.19	0	−0.73^**^	0.37	−0.34	0.13	0.12	1								
PPO	−0.04	−0.02	0.08	−0.17	0.1	0.32	−0.28	−0.21	1							
K	−0.47^*^	−0.57^**^	−0.42	0.48^*^	−0.05	−0.50^*^	0.13	0.4	−0.35	1						
Na	−0.17	−0.26	−0.41	0.49^*^	−0.27	0.32	0.34	0.17	0.04	0.21	1					
Ca	−0.54^*^	−0.48^*^	0.17	−0.2	0.09	−0.31	−0.11	−0.42	0.23	−0.05	0.12	1				
Mg	−0.34	−0.4	−0.27	0.43	0	0.01	0.19	−0.06	−0.17	0.37	0.58^**^	0.13	1			
TP	−0.80^**^	−0.72^**^	0.19	0.03	−0.01	−0.03	−0.01	−0.31	0.08	0.18	0.31	0.56^*^	0.29	1		
TF	−0.48^*^	−0.53^*^	−0.27	0.19	−0.15	0.21	−0.25	0.04	0.04	0.09	0.59^**^	0.42	0.45^*^	0.56^**^	1	
TT	0.02	0.14	0.49^*^	−0.60^**^	0.4	−0.14	−0.11	−0.25	−0.12	−0.09	−0.15	0.1	−0.28	0.12	0.18	1

T, temperature; P, precipitation; S, sunlight; TP, total polysaccharides; TF, total flavonoids; and TT, total triterpenoids.

^*^Difference level at 0.05.

^**^Difference level at 0.01.

### Relationship among various responsive physiological indices

Physiological responses to changeable environment directly or indirectly cause variances in secondary metabolite accumulation. Among four kinds of mineral nutrients and three kinds of secondary metabolite accumulation, Ca and Mg were positively correlated with polysaccharide and total flavonoid accumulation without fertilization conditions (Table 5), which was inconsistent with the findings of Deng *et al.* (2019), who reported that Ca and Mg had a significant negative correlation with total flavonoid accumulation under five nitrogen fertilization levels (Deng *et al.*, 2019). This discrepancy could be caused by nitrogen availability, which influences the absorption and distribution of mineral nutrients, further affecting secondary metabolite accumulation in *C. paliurus* (Deng *et al.*, 2019).

Environmental factors have an influence on physiological activities, in turn, various physiological activities are together involved in adapting changeable environment. K and Ca in leaf clustered into one group (Table 5, Fig. 8C and D) inferred that they could act on similar biological processes (Coskun *et al.*, 2013; Gattward *et al.*, 2012), including the promotion of osmotic protection, or the inhibition of leaf water loss via stomatal regulation (Ahmad and Maathuis, 2014; Peiter, 2011). In addition, four kinds of mineral nutrient were grouped with antioxidant enzymes (Table 5, Fig. 8C and D). For example, Mg had a positive relationship with SOD and CAT, especially the group was formed between Mg and CAT (Table 5; Fig. 8C and D). Mg directly or indirectly participates in biological processes in plants, such as the synthesis of chlorophyll (Masuda, 2008) and Ribulose-1,5-bisphosphat-carboxylase/-oxygenase activity (Portis, 2003); but Mg deficiency reduces the absorption and utilization of light energy, resulting in the production of ROS and the increase of antioxidant enzyme activity (Guo *et al.*, 2016).

### Homological relationship between leaf morphology and SSR analysis

Plants exhibit substantial genotypic diversity during the developmental processes. Leaves are one of the most visible and vital organs, with genotypic variations in shape, colour, margin and texture (Bruno *et al.*, 2008), and this valuable morphological information can help identify species (Arturo *et al.*, 2015; Kala and Viriri, 2018). In this study, leaves from the six provenances differed in shape, colour and area. However, the leaves from FJ and AJ had many similar characteristics, suggesting that their origin could be similar, which was further manifested by the results of the genetic relationship analysis.

The relatively high number of alleles generated by the SSR markers demonstrates the usefulness of the marker system for the detection of genetic diversity (William *et al.*, 2020). Li *et al.* (2017) reported that 24 alleles were detected using six SSR markers. In our study, the number of alleles investigated ranged from 3 to 14, and the mean number was 8.6, which was significantly higher than the 3.83 alleles/locus reported by Li *et al.* (2017). This indicated that there was a good level of allelic diversity. Meanwhile, the mean gene diversity obtained in our study was different from the findings of Li *et al.* (2017), who reported a gene diversity of 0.09 among 26 natural provenances. This discrepancy could be due to the selection of different SSR markers. These results suggest that an SSR analysis is an efficient method for analysing genetic relationships in plants.

Homology relationships are important for understanding the evolutionary relationships among different genotypic resources and could facilitate breeding and conservation programs. In this study, according to the homology relationships, we found two groups (Fig. 7) with a coefficient of 0.51. The first of which contained only WF, and the second group included FJ, AJ, JH, JX and TG distributed in the Fujian, Zhejiang, Hubei and Jiangxi Provinces. These relationships are consistent with the results of Li *et al.* (2017), who reported that *C. paliurus* appeared to be an expanding species in subtropical China, but less genetic differentiation and a high gene flow occurred among natural populations of *C. paliurus* distributed in the Fujian, Zhejiang and Jiangxi Provinces, explaining why they were clustered into a larger group. Similar observations have been made in other species (Bhattarai and Mehlenbacher 2017; Dettori *et al.*, 2015; Zhu *et al.*, 2016; Liang *et al.*, 2018; Patzak *et al.*, 2020; Suvi *et al.*, 2020). This confirmed that *ex situ* conservation benefits the preservation of this species’ gene pool and maintains regional differences in diversity.

## Conclusions

Plants of the six *Cyclocarya* provenances were conserved at a resource plantation in Quanzhou, Fujian Province, and studied variations in plant growth and leaf physiological response to environmental factors during the growth period and further analysed homological relationships by leaf morphological characteristics and SSR. The results showed that (i) plants of *C. paliurus* from the six provenances varied in growth; (ii) physiological changes during the growth period had differences in WC, TSS content, mineral content, antioxidant enzyme activity and secondary metabolite accumulation; (iii) variation in plant growth and physiological performances had significant relation with environmental factors, especially temperature and precipitation; (iv) leaf morphology among the six provenances differed in shape, colour and area. Moreover, two groups were clustered at a coefficient of 0.51 by SSR analysis, of which one contained only WF and the other included FJ, AJ, JH, JX and TG distributed in the Fujian, Zhejiang, Hubei and Jiangxi Provinces. The results of this study provide information on physiological response to environmental factors at a resource plantation of *ex situ* conservation and benefit to selecting suitable provenances for *Cyclocarya* cultivation.

## Funding

This work was supported by the Fujian Province Science and Technology Project (grant number 2017N0028), Quanzhou Science and Technology Project (grant number 2018N007) and The Program for Excellent Talents in Quanzhou City (grant number 2021C043R).

## Supplementary material

Supplementary material is available at *Conservation Physiology* online.

## Data availability

The data sets used and/or analysed during the current study are available from the corresponding author on request.

## Conflict of Interest

The authors declare that they have no competing interests.

## Supplementary Material

Web_Material_coac016
