# Supplementary material for: Plant growth, physiological variation and homological relationship of Cyclocarya species in ex situ conservation
Source: Conserv Physiol. 2022 May 7;10(1):coac016. doi: 10.1093/conphys/coac016 (PMC9082347; doi:10.1093/conphys/coac016)
Supplement: Web_Material_coac016 [file web_material_coac016.docx]

**Table S1.** Environmental parameters on six *Cyclocarya* provenances

| Provenance | N | E | MAT(℃) | AP(mm) | AS(h) |
| --- | --- | --- | --- | --- | --- |
| FJ | 25°19′19″ | 118°17 ′39″, | 20±2 | 1500-2000 | 4000-4500 |
| AJ | 30°14′24″ | 119°22′48″ | 19±2 | 1300-1700 | 2100-2200 |
| WF | 30°6′36″ | 110°31′48″ | 15±2 | 1600-1700 | 1500-1600 |
| TG | 28°31′48″ | 114°22′12″ | 16±2 | 1700-1800 | 1400-1500 |
| JX | 26°18′36″ | 114°30′00″ | 14±2 | 1800-1900 | 1500-1600 |
| JH | 26°22′12″ | 108°22′48″ | 16±1 | 1200-1300 | 1200-1300 |

N means latitude; E means longitude; MAT means mean annual temperature; AP means annual precipitation; AS means annual sunlight; FJ, Yongchun, Fujian Province; JX, Jinggangshan, Jiangxi Province; AJ, Anji, Zhejiang Province; TG, Tonggu, Jiangxi Province; JH, Jianhe, Guizhou Province; WF, Wufeng, Hubei Province.
